# Bioactive Earthworm Peptides Produced by Novel Protease-Producing *Bacillus velezensis* PM 35 and Its Bioactivities on Liver Cancer Cell Death *via* Apoptosis, Antioxidant Activity, Protection Against Oxidative Stress, and Immune Cell Activation

**DOI:** 10.3389/fmicb.2022.892945

**Published:** 2022-08-10

**Authors:** Pimphan Wasunan, Chutamas Maneewong, Wichittra Daengprok, Mongkol Thirabunyanon

**Affiliations:** ^1^Program in Biotechnology, Faculty of Science, Maejo University, Chiang Mai, Thailand; ^2^Program in Food Science and Technology, Faculty of Engineering and Agroindustry, Maejo University, Chiang Mai, Thailand

**Keywords:** *Amynthas arenulus*, antioxidant, immune, liver cancer, oxidative stress, peptides, protease

## Abstract

Earthworms have long been used as traditional medicine. The purposes of this research were to create bioactive peptides from the unique *Amynthas arenulus* earthworm (PAAEs) and test their potentials on liver cancer bioprophylactic activity, antioxidant, oxidative stress protection, and immune cell activation. This earthworm had a high protein content ratio, at 55.39%. Besides, PM 35 is one out of 58 bacteria isolated from the earthworm carcasses that exhibited the highest protease and yield protein production which was chosen as the protease-producing bacteria to hydrolyze the protein. The genera were identified by 16S rRNA and 16S–23S rRNA comparison and confirmed as *Bacillus velezensis* PM 35. The response surface methodology was applied to optimize these hydrolysis parameters, i.e., the enzyme/substrate (E/S) concentration ratio [1%–3% (v/v)] and time (1–3 h) of the hydrolyzing earthworm’s proteins. The optimal hydrolyzing conditions were 3% (v/v) of E/S concentration ratio and 3 h of hydrolysis time, which found protein-hydrolysate yield (24.62%) and degree of hydrolysis (85.45%) as the highest. After being challenged in the gastrointestinal tract-resistant model, these PAAEs (MW <3 and 3–5 kDa) induced liver cancer cell (HepG2) death *via* apoptotic action modes (cell morphological change and DNA fragmentation). The PAAEs (MW <3 kDa) exhibited significant antioxidant activity *via* DPPH, ABTS, and FRAP with IC_50_ values of 0.94, 0.44, and 6.34 mg/ml, respectively. The PAAEs (MW < 3 kDa) were non-cytotoxic and protected the mouse fibroblast cells (L929) against oxidative stress. These PAAEs (MW < 3 kDa, 0.2 mg/ml) stimulated the B lymphocytes (122.3%), and T lymphocytes (126.7%) proliferation. This research suggests that PAAEs can be used in a variety of applications, especially in the food and pharmaceutical industries.

## Introduction

Hepatocellular carcinoma is the third highest cause of cancer-related mortality worldwide (Sung et al., 2021). Chronic hepatitis B virus (HBV) and/or hepatitis C virus (HCV) infection is the primary causes (Parkin, 2006). Cirrhosis, which is caused by excessive alcohol intake over an extended period, has been related to the development of this cancer (La Vecchia et al., 1998). Tobacco usage, aflatoxin-contaminated food, hemochromatosis (Stuver and Trichopoulos, 2008), obesity (Seitz and Stickel, 2006), and diabetes are all established risk factors. Hepatocellular carcinoma can be treated with chemotherapy, radiation, surgery, immunotherapy, and other therapies. Bioactive components such as peptides have also been employed to treat hepatocellular carcinoma (Wang et al., 2016).

Bioactive peptides are organic molecules composed of 2–20 amino acids linked together by peptide bonds, with the possibility of more than 20 amino acid residues. Bioactive peptides have been shown to have antioxidant (Xia et al., 2019), antihypertensive (Shobako et al., 2018), antimicrobial (Tejano et al., 2019), immunomodulatory effects (Yang et al., 2020), and anti-inflammatory activity (Xie et al., 2018). Plant and animal peptides have anticancer properties. For example, rice bran peptides have been shown to inhibit colon and liver cell proliferation (Kannan et al., 2008). Tilapia peptides have been demonstrated to inhibit proliferation of adenocarcinoma cells (HeLa), hepatocellular carcinoma cells (HepG2), fibrosarcoma cells (HT1080), Cercopithecus aethiops kidney cells (COS-7), and human kidney cells (WS-1; Chang et al., 2011a). Spotted babylon snail peptides prevent the development of adenocarcinoma colon cells (Caco-2; Petsantad et al., 2020).

Earthworms are invertebrates that have been used for centuries as a traditional medicine (Chen et al., 2016). Earthworms have been shown to have medicinal properties. In the genus *Lampito*, *Eudrilus*, *Eisenia*, *Perionyx*, *Metaphire*, and *Lumbricus*, earthworm extracts such as earthworm paste, earthworm powder, coelomic fluid, and fibrinolytic enzyme, have been found to have anti-inflammatory (Mathur et al., 2011), antioxidant (Balamurugan et al., 2008), and antimicrobial (Wang et al., 2003) properties. Furthermore, these earthworms can suppress cancer cells such as oral cancer cells (Augustine et al., 2017), HeLa cells (Yanqin et al., 2007), breast cancer cells (Liu et al., 2017), prostate cancer cells, and colorectal cancer cells (Shafi and Faleh, 2019).

Furthermore, the genus *Amynthas* earthworms have been revealed to regenerate axons in peripheral nerve damage (Chen et al., 2010), stimulate Schwann cells (Chang et al., 2011b), have antiasthmatic properties (Chen et al., 2016), and stimulate osteoblast activity (Fu et al., 2014). Only 26 species of *Amynthas* earthworms have been recognized in Thailand (Jeratthitikul et al., 2017). *Amynthas arenulus* is a newly discovered earthworm that inhabits the highland paddy system, modified from the dipterocarp forest of northeast Thailand (Bantaowong et al., 2014). However, the *A. arenulus* earthworm explored in this study has not yet been reported earlier to against cancers. Thus, the present research aims to produce bioactive peptides from *A. arenulus* earthworm which are hydrolyzed by highly efficient protease-producing bacteria and have their potential as liver cancer bioprophylactic, antioxidant activity, protect against oxidative stress-induced cells, and immune cell activation.

## Materials and Methods

### Earthworm Collection

The mature earthworms were obtained in the northeast province of Nakhon Phanom, Thailand (Lat. 17°30′31.4352″ N; Long 104°6 0.27″ E). Individual earthworms were weighed between 11.6 and 13.5 g. The external morphology was observed to have 176 segments, 267–465 mm of body length, 13.3 mm of body width, spermathecal pores paired in segments 6/7–8/9, the first dorsal pore in segment 12/13, the clitellum annular in setae 14–16, and male pores paired in segment 18 (Bantaowong et al., 2014).

### Earthworm Identification

DNA was extracted from the clitellum of an adult earthworm (Mathur et al., 2010). Total genomic DNA was extracted using a genomic DNA mini kit (Tiangen, Taiwan). The mitochondria cytochrome c oxidase I (COI) gene was employed using the universal forward primer (LCO1490F; 5′-GGTCAACAAATCATAAAGATATTGG-3′) and reverse primer (HCO2198R; 5′-TAAACTTCAGGGTGACCAAAAAATCA-3′; Folmer et al., 1994). The PCR was operated in a total volume of 50 μl containing 25 μl of DreamTaq Green PCR Master Mix (2X; Thermo Fisher Scientific, Lithuania), 5 μl of each primer (5 μM; Integrated DNA Technologies, Singapore), 5 μl of genomic DNA, and nuclease-free water (Thermo Fisher Scientific, Lithuania) up to 50 μl. The PCR cycling conditions were performed with an initial denaturation step at 95°C for 3 min, followed by 35 cycles of denaturation at 95°C for 30 s, an annealing temperature of 52°C for 30 s, and extension at 72°C for 1 min, followed by a final extension step at 72°C for 15 min. The PCR product was analyzed on a 1.5% agarose gel containing ethidium bromide (10 mg/ml). PCR yield was purified using a TAINquick mini purification kit (Tiangen, Taiwan). The sequence was analyzed by comparing it to a BLAST search in the GenBank database for the COI sequence.^1^

### Preparation of Dried Earthworm Powders

The digestive systems of mature earthworms were removed after they were rinsed with running tap water to eliminate dirt from the body surface. The earthworms were thoroughly washed using sterile distilled water. Earthworms were pulverized and stored at −20°C (Anitha and Jayraaj, 2012).

### Preparation of Earthworm Protein Isolates

Earthworm powder was defatted using a hexane solution (Sivaramakrishnan and Incharoensakdi, 2017). Briefly, earthworm powder, distilled water, and 99% of hexane solution (Merck, Darmstadt, Germany) were combined in a separatory funnel in the ratio of 1:10:10 (w/v/v). The mixture was gently inversed approximately 300 times. The protein fraction in the water layer was separated, and then, the water was evaporated in an incubator at 50°C for 24 h. The proteins were extracted from the defatted sample by isoelectric precipitation (Lee et al., 2016). Briefly, degreased proteins were extracted using alkaline solution (pH 10.0) at the ratio of 1:10% (w/v) at 50°C for 1 h using a magnetic stir bar. Consequently, the reaction mixture was centrifuged at 2,700 *g* in 4°C to separate the supernatant. The proteins were isolated from alkaline soluble through acid precipitation. The pH was readjusted by addition of HCl to pH 4.0 to near the isoelectric point of proteins. The suspension was incubated at 50°C for 1 h using a magnetic stir bar. The suspension was centrifuged at 2,700 *g* in 4°C. After centrifugation, protein precipitates by alkaline solubilization and acid precipitation were additionally washed with sterile distilled water by centrifugation at 2,700 *g* in 4°C. The washed earthworm protein isolates were lyophilized.

### Protein Analysis of Earthworm Protein Isolates

The earthworm protein isolates were determined by protein analysis according to the established methodology in AOAC 2000 (William, 2000). For protein analysis, samples were submitted at the total Kjeldahl nitrogen process. The percentages of nitrogen and protein were estimated using Eqs. (1) and (2). The nitrogen concentration was converted to protein concentration using a conversion factor of 6.25 (equal to 0.16 g of nitrogen per gram of protein; Zakaria et al., 2013).


(1) $$\%\mathrm{Nitrogen}=\left[\frac{\left(V_{\mathrm{sample}}-V_{\mathrm{blank}}\right)x\;0.1\;N\;HCl\;x\;1.4007}{\mathrm{Weight of sample}\;\left(g\right)}\right]x100$$

(2) $$\%\mathrm{Protein}=\left(\%\mathrm{Nitrogen}\right)\;x\;\mathrm{protein factor}$$

### Protease-Producing Bacteria Screening and Protease Production

#### Bacteria Isolation

Earthworm carcasses and dried earthworms were separately washed and soaked in sterile distilled water. Bacteria were isolated from water in either the earthworm carcass or dried earthworm process. The earthworm carcass lavage was 10-fold diluted with 0.85% sodium chloride, spread on nutrient agar (NA) media (Criterion, United States), and incubated at 37°C for 24 h. Single colonies appeared and were chosen. Colonies were cultured in nutrient broth (NB) media (Criterion, United States), incubated at 37°C for 24 h, and stored with 20% glycerol at −20°C. Dried earthworms were soaked with sterile distilled water at 1.0% (w/v) and incubated at 37°C, 120 rpm for 24 h. Following soaking and incubation, water was diluted 10-fold with 0.85% NaCl and dispersed over the NA media. Bacteria were cultured at 37°C for 24 h. Single colonies appeared and were chosen. Colonies were grown in NB media and incubated at 37°C for 24 h. It was kept at −20°C with 20% glycerol until it was used in further research.

#### Production of Bacterial Protease

##### Screening of Protease-Producing Bacteria

The 58 isolates of protease-producing bacteria were carried out utilizing the agar well diffusion technique (Rahman et al., 2018). Bacteria that had been stored were grown in NB media and incubated at 37°C for 24 h. The bacteria (10 μl) were then introduced to skim milk agar (SMA; Oxoid, England) media and cultured at 37°C for 72 h to examine the protease-producing zone on the media.

##### Crude Protease Preparation

Crude protease was prepared using skim milk media (Rupali, 2015). The isolated protease was cultured in skim milk broth media at 37°C for 72 h. To eliminate undesirable particles, the bacterial cultures were centrifuged at 13,710 *g* for 5 min at 4°C. The clear supernatant (crude protease) that was subjected to subsequent purification was stored for future research.

##### Determination of Protein

The protein was determined using the Lowry technique (Lowry et al., 1951). The standard was bovine serum albumin fraction V (PAA Laboratories, Austria).

##### Protease Activity Assay

The substrate for this experiment was 1.0% (w/v) casein (Sigma, United States). One hundred microliter of enzyme was combined with 100 μl of 1.0% casein (w/v) in 0.05 M potassium phosphate buffer (pH 8.0) and incubated at 37°C for 20 min. After the incubation period, the reaction was stopped using 0.2 ml of 0.4 M trichloroacetic acid (TCA, AppliChem GmbH, Darmstadt, Germany), which was incubated for 15 min at room temperature. The sample was then centrifuged at 9,520 *g* for 10 min to remove the pellets. From this, 0.2 ml of supernatant was mixed with 0.5 ml of 0.4 M sodium carbonate and incubated at room temperature for 30 min with 0.1 ml of 1 N Folin–Ciocalteu’s phenol solution (Merck, Germany; Rupali, 2015). The absorbance at 660 nm was measured with a spectrophotometer. Under the standard assay conditions, one unit of protease activity was defined as the quantity of protease generated, which was estimated and represented in micrograms of tyrosine released.

##### Purification of Protease Enzyme

Acetone was used to precipitate protease enzyme purification (Shaikh et al., 2018). Protease enzyme was precipitated in the bacterial supernatant with an 80% cold acetone solution in a 1:4 (v/v) ratio at 20°C overnight. The suspension was centrifuged at 1,520 *g* at 4°C for 30 min. The pellets were dissolved in cold acetone and recentrifuged to recover the residual crude enzyme. After being stored at −80°C, the crude enzyme was lyophilized.

### Identification of Protease-Producing Bacteria

Bacteria were cultured in NB and incubated at 37°C for 24 h. The bacterial suspension was transferred to 1.5-ml microcentrifuge tube and centrifuged at 13,710 *g*, 4°C for 5 min. The genomic DNA mini kit (Tiangen) was used to extract the DNA pellet. Extracted DNA was determined and quantified by measuring at absorbance 260/280 nm with Nanodrop 2000/2000 C spectrophotometer V1.0 (Thermo Scientific, United States).

To amplify bacterial genomic DNA, two pairs of primers were utilized (Thirabunyanon and Thongwittaya, 2012). For the first pair, the 16S rRNA region was employed using a universal forward primer (27F; 5′-AGAGTTTGATCCTGGCTCAG-3′) and a reverse primer (1494R; 5′-CTACGGCTACCTTGTTACGA-3′; Integrated DNA Technologies; Lane, 1991). The PCR conditions were performed using DreamTaq Green PCR Master Mix (2X; Thermo Fisher Scientific), which included an initial denaturation step at 95°C for 3 min, 35 cycles of denaturation at 95°C for 30 s, annealing of 50°C for 30 s, and extension at 72°C for 1 min followed by final extension step at 72°C for 15 min. It was carried out in a final volume of 50 μl reaction mixture including 25 μl of DreamTaq Green PCR Master Mix (2X), 5 μl of each primer (5 μM), 5 μl of genomic DNA, and 10 μl of nuclease-free water. For the second pair, the 16S–23S rRNA internal transcribed spacers using the forward S-D-Bact-1494-a-S-20F (5′-GTCGTAACAAGGTAGCCGTA-3′) and reverse L-D-Bact-0035-a-A-15R (5′-CAAGGCATCCACCGT-3′) primers (Integrated DNA Technologies; Daffonchio et al., 1998). The PCR conditions included an initial denaturation step at 95°C for 3 min, 30 cycles of denaturation at 95°C for 30 s, annealing of 55°C for 30 s, and extension at 72°C for 1 min followed by final extension step at 72°C for 15 min. It was carried out in a final volume of 50 μl reaction mixture including 25 μl of DreamTaq Green PCR Master Mix (2X), 5 μl of each primer (5 μM), 5 μl of genomic DNA, and 10 μl of nuclease-free water.

The PCR product was analyzed on a 1.5% agarose gel containing ethidium bromide (10 mg/ml). PCR yield was purified using a TAINquick mini purification kit (Tiangen). The sequence was analyzed by doing a BLAST search in the GenBank database for the 16S rRNA region and 16S–23S rRNA internal transcribed spacers sequence.^1^

### Hydrolysis Design for Earthworm Protein Isolates

The powder of earthworm protein isolates was weight 1% (w/v) in potassium phosphate buffer (pH 8.0). The protein suspension was hydrolyzed with protease enzyme (1 mg/ml of stock enzyme concentration) at 120 rpm, temperature 35°C, various enzyme/substrate (E/S) concentration ratio [1%–3% (v/v)], and time to hydrolyse (1–3 h).

The central composite design (CCD) was conducted in the optimum condition of the significant factors for the protein hydrolysis under response surface methodology (RSM; Rodrigues et al., 2017). In this experimental design, optimization is handled by varying two factors: the E/S concentration ratio [A: 1%–3% (v/v)] and the time of this hydrolysis (B: 1–3 h). Table 1 shows the experimental variables and levels of the optimize experiment. The CCD code values of each variable in each treatment are presented in Table 2. The protein hydrolysate was weighed to evaluate the yield of each hydrolyse, as well as the protein hydrolysate determined the degree of hydrolysis in further assay. The data were also analyzed using the Design-Expert program version 11 from Stat-Ease, Inc., Minneapolis, United States.

**Table 1:** Variables, levels, and ranges of operating factors for optimization.

Operating factors	Coding	Units	Levels and ranges		
			Low (−)	Center point (0)	High (+)
E/S Concentration ratio	A	% v/v	1	2	3
Time	B	Hour	1	2	3

**Table 2:** Treatments generated based on the central composite design to hydrolysis conditions.

Treatments	A; Enzyme/substrate concentration ratio	B; Time
1	+1	+1
2	0	+1
3	+1	0
4	0	0
5	0	−1
6	−1	0
7	−1	+1
8	0	0
9	−1	−1
10	0	0
11	+1	−1

### Degree of Hydrolysis

Trichloroacetic acid was used to determine the degree of protein hydrolysis. The protein hydrolysates (500 μl aliquots) were combined with 500 μl of 20% of TCA solution to achieve final 10% TCA concentration (Silvestre et al., 2013). The mixture was incubated at room temperature for 30 min before being centrifuged at 2,700 *g* for 15 min at 25°C. The supernatant was determined using the Lowry method (Lowry et al., 1951). The Eq. (3) was used to calculate the degree of hydrolysis:


(3) $$\mathrm{Degree of hydrolysis}\;\left(\%\right)=\frac{\mathrm{Soluble protein content in}\;10\%\;\mathrm{of}\;TCA\;\left(\mathrm{mg}\right)}{\mathrm{Total protein content}\;\left(\mathrm{mg}\right)}\;x\;100$$

### Producing Peptides Resistant to Simulated Gastrointestinal Environment

#### Simulated Gastric Environment

The simulated gastric solution was prepared by dissolving 0.2 g of sodium chloride in 100 ml of deionized water. The pH was adjusted to 2.0 with 1 N HCl. The 0.32% (w/v) of pepsin enzyme (Sigma, United States) was added and stirred at 37°C. The protein hydrolysates were examined with simulated gastric solution by constant shaking at 120 rpm, 37°C for 2 h. The enzyme was then inactivated by adjusting pH to 7.2 with 1 N NaOH. The mixture was subsequently centrifuged at 2,700 *g* for 30 min at 4°C to obtain soluble protein hydrolysates resistant to the simulated gastric environment (Kannan et al., 2008). The gastric environment-resistant earthworm protein hydrolysates were then lyophilized.

#### Simulated Intestinal Environment

The simulated intestinal solution was created as follows: 0.68 g of potassium phosphate monobasic and 7.7 ml of 0.2 N NaOH were dissolved in 100 ml of deionized water. The pH was adjusted to 8.0 with 1 N NaOH. The 0.1% (w/v) of pancreatin enzyme (Sigma, United States) was added, stirred for 30 min, and the temperature was then maintained at 37°C. The gastric environment-resistant earthworm protein hydrolysates were treated with simulated intestinal solution by constant shaking at 120 rpm, 37°C for 2 h. The enzyme was inactivated by heating at 85°C for 10 min. The protein hydrolysates were centrifuged at 2,700 *g* for 30 min at 4°C to obtain protein hydrolysates resistant to the simulated intestinal environment (Kannan et al., 2008). The intestinal environment-resistant earthworm peptides were then lyophilized.

### The Fractionation of Gastrointestinal-Resistant Peptides by Ultrafiltration

An ultrafiltration system was used for the fractionation of gastrointestinal-resistant peptides from *A. arenulus* earthworms (PAAEs). The filtered soluble PAAEs were run through sequential ultrafiltration columns with membrane cartridges having nominal molecular weight cutoffs (MWCO) of 50, 30, 10, 5, and 3 kDa (GE Healthcare Bio-Sciences AB, Sweden). The PAAEs were first permeated through the 50 kDa membrane, followed by 30, 10, 5, and then 3 kDa MWCOs, respectively. The resulting retentates from each of the MWCO were lyophilized and stored at −20°C.

### Cell Line and Growth Conditions

Liver cancer cells (HepG2) and normal mouse fibroblast cells (L929) were purchased from the American Type Culture Collection (ATCC), which were used in this study. These cells were maintained in Dulbecco’s modified Eagle medium (DMEM) supplemented with 10% heated inactivated fetal bovine serum (FBS), 1% MEM non-essential amino acids (100x MEM NEAA), and 1% penicillin–streptomycin (10,000 U/ml penicillin and 10,000 μg/ml streptomycin; all from Gibco, United States). Cells were cultured in a 25 cm^3^ flask (Nunc, Denmark) and incubated in a CO_2_ incubator (Forma Scientific, 3,111, United States) at 37°C with 5% CO_2_ and 95% humidity. These cell lines were subsequently employed to examine the cellular activities.

### Proliferative Inhibition of Liver Cancer Cells

The MTT method was used to examine the effect of PAAEs on cellular proliferation. This method is associated with mitochondrial dehydrogenase’s ability to convert 3-(4,5-dimethylthiazolyl-2-yl)-2,5-diphenyltetrazolium bromide (MTT; Sigma, United States) into purple formazan. Briefly, 100 μl of exponential phase trypsinized HepG2 cells was grown in 96-well flat-bottom plates (Nunc, Denmark) at a concentration of 10^4^ cells/ml and incubated overnight at 37°C with 5% CO_2_ and 95% humidity. After 24 h of incubation, 100 μl of PAAEs (concentrations ranging from 0.02 to 0.2 mg/ml) was added to the 96-well plate and then incubated. Untreated cells served as the control. Ten microliters of MTT [0.5 mg/ml in dimethyl sulfoxide (DMSO)] was used and incubated for 4 h. To solubilize formazan crystals, 100 μl of DMSO (Sigma, United States) was added for 10 min. Absorbance was read at 595 nm (A_595_) using a microplate reader (Thirabunyanon and Hongwittayakorn, 2013). The growth inhibition rates were expressed as percentages and calculated with following Eq. (4). All experiments were performed in triplicate. Half the maximal inhibitory concentration (IC_50_) of PAAEs was determined using GraphPad Prism software version 9.0.0 from California, United States.


(4) $$\mathrm{Antiproliferation}\;\left(\%\right)=\left(1-\frac{\mathrm{Abs}.\;\mathrm{of treated cells}}{\mathrm{Abs}.\;\mathrm{of control}\;\left(\mathrm{untreated}\right)\;\mathrm{cells}}\right)x\;100$$

### Cytotoxicity of Normal Mouse Fibroblast Cells

The exponential phase of L929 cells (10^4^ cells/ml) was seeded into 96-well flat-bottom plates and incubated at 37°C with 5% CO_2_ and 95% humidity. After 24 h of incubation, cells (100 μl) were treated with the IC_50_ values of PAAEs (100 μl) and incubated. Untreated cells served as the control. Ten microliters of MTT (0.5 mg/ml in DMSO) was added and incubated for 4 h. Subsequently, 100 μl of DMSO was added to the dissolve formazan crystals for 15 min (Hu et al., 2019). Absorbance was read at 595 nm (A_595_) using a microplate reader. The viability rates were expressed as percentages in following Eq. (5).


(5) $$\mathrm{Viability rate}\;\left(\%\right)=\left(\frac{\mathrm{Abs}.\;\mathrm{of treated cells}}{\mathrm{Abs}.\;\mathrm{of control}\;\left(\mathrm{untreated}\right)\;\mathrm{cells}}\right)x\;100$$

### Morphological Changes Under Microscope and Dual AO/PI Staining

#### Morphological Changes Under an Inverted Microscope

Five milliliters of HepG2 cells in exponential phase (10^4^ cells/ml) was grown in 12-well flat-bottom plates (Nunc, Denmark) under proper conditions. Cells were evaluated with 5 ml of IC_50_ values of PAAEs. DMSO [0.5% (v/v)] was used as a positive control. The supernatants and non-adhesive cells were discarded after 24 h and rinsed with PBS. The morphological changes in the HepG2 cells that were treated with PAAEs were investigated and compared with control (untreated cells) using an inverted microscope (Olympus, Japan; Amini-Sarteshnizi et al., 2014).

#### Visualization of Morphological Apoptotic Changes by Dual AO/PI Staining

Apoptosis was examined by staining with acridine orange (AO; Sigma, United States) and propidium iodide (PI; Sigma, United States; Fani et al., 2015; Srikham and Thirabunyanon, 2022). HepG2 cells (10^4^ cells/ml) were initially grown and incubated for 24 h in an 8-well chamber slide system (Nunc, Denmark). The cells were subsequently given an IC_50_ of PAAEs. After 24 h of incubation, cells were washed twice with PBS to remove the media. The morphological features were stained using a 1:1 AO/PI mixture consisting of 10 μg/ml of AO and 10 μg/ml of PI (dissolved in PBS). The morphological changes were detected using a fluorescence inverted microscope (Olympus, Japan). Green cells show viability, whereas red fluorescence signals late apoptosis, owing to condensed and damaged chromatin, respectively.

### DNA Fragmentation

This study was carried out according to the method previously described, with slight modifications (Srikham et al., 2021). Trypsinized HepG2 cells (5 ml) were cultured in 12-well flat-bottom plates (Nunc) at a concentration of 10^4^ cells/ml and incubated under appropriate conditions. Following incubation, cells were treated with 5 ml of IC_50_ values of PAAEs and incubated for 24 h. DMSO (0.5% v/v) served as the positive control. Cells were detached and centrifuged at 13,710 *g* for 5 min to harvest adhesive cells. PBS solution was then used to wash the pelleted cells. After that, DNA was retrieved from cell pellets using a DNA extraction kit (Tiangen). The extracted DNA was electrophoresed using a 1.5% agarose gel, and the results were subsequently visualized under UV light (UltraSlim LED illuminator, California, United States).

### Determination of Antioxidant Activity

#### 2,2-Diphenyl-1-Picrylhydrazyl Radical Scavenging Activity Assay

PAAEs were carried out using DPPH radical-scavenging activity (Petsantad et al., 2020; Chit-aree et al., 2021). The DPPH (Sigma, United States) radical solution (0.1 mM) was prepared in methanol. PAAEs were generated at concentrations ranging from 0.02 to 0.2 mg/ml. The PAAEs were loaded onto the DPPH radical solution in a 1:4% (v/v) ratio, i.e., 25 μl of the PAAEs: 100 μl of DPPH radical solution. After being made, the mixture was vortexed and incubated at room temperature for 30 min in the dark. Tocopherol served as the positive control, and absorbance at 517 nm (A_517_) was measured using a microplate reader (SPECTRO Star Nano, BMG Labtech, Germany).

#### 2,2′-Azino-Bis (3-Ethylbenzothiazoline-6-Sulfonic Acid) Radical Scavenging Activity Assay

PAAEs were performed by using ABTS radical cation decolorization activity (Petsantad et al., 2020). Seven millimolar of ABTS (Sigma, United States) solution was mixed with 2.45 mM of potassium persulfate (Ajax Finechem, Australia) and incubated at room temperature for 12–16 h in darkness to create an ABTS cation radical. The ABTS cation radical was diluted with methanol to achieve an absorbance of 0.700 ± 0.02 at 734 nm. PAAEs were produced at concentrations ranging from 0.02 to 0.2 mg/ml. The PAAEs were combined with the ABTS cation radical in a 1:30% (v/v) ratio, i.e., 10 μl of the PAAEs: 300 μl of ABTS cation radical solution. The mixture was incubated at room temperature for 10 min in the dark. Tocopherol was used as the positive control, and absorbance at 734 nm (A_734_) was measured using a microplate reader.

#### The Ferric Reducing Antioxidant Power Assay

This method investigated the ability of antioxidant to reduce ferric iron, with modification of Farsani et al. (2021). PAAEs were prepared at concentrations ranging from 0.02 to 0.2 mg/ml. In the beginning, 10 ml of acetate buffer pH 3.6 was combined with 1 ml of 10 mM of TPTZ [(2,4,6-tri(2-pyridyl)-1,3,5-triazine); Sigma, Switzerland] and 1 ml of 20 mM of ferric chloride (Ajax, Australia). PAAEs were mixed with FRAP at the ratio of 1:30 (v/v). The mixture was then incubated at room temperature for 10 min. Tocopherol served as the positive control, and absorbance at 593 nm (A593) was measured using a microplate reader.

#### Calculations of Percentage Inhibition

To determine the ability to scavenge activity and ferric reducing antioxidant power following Eq. (6) was employed, where A_control_ denotes the absorbance of the control (no PAAEs), and A_sample_ denotes the absorbance of the PAAEs.


(6) $$\mathrm{Scavenging activity}\;\left(\%\right)=\left[\frac{\left(A_{\mathrm{Control}}-A_{\mathrm{sample}}\right)}{A_{\mathrm{control}}}\right]x\;100$$

### Protective Effect of PAAEs on the Viability of Oxidation-Induced Cells

An *in vitro* experiment model was assessed the protective effect of PAAEs against oxidative damage induced by hydrogen peroxide (H_2_O_2_; Merck, United States; Craciunescu et al., 2013). Mouse fibroblast cells were grown with completed media under proper conditions. Cells (100 μl) were seeded at a density of 10^4^ cells/ml in a 96-well flat-bottom plate and incubated overnight. Cells simultaneously were treated with the IC_50_ values of PAAEs + H_2_O_2_ (final conc. 50 μM) and H_2_O_2_ (final conc. 50 μM) and then incubated for 24 h. The H_2_O_2_ served as comparative group. The viability of the cells was investigated using 10 μl of MTT solution (0.5 mg/ml in DMSO) for 4 h. Finally, 100 μl of DMSO was added to solubilize the formazan crystals for 10 min. The formazan level was assessed by measuring absorbance at 595 nm with a microplate reader (SPECTRO Star Nano, BMG Labtech, Germany). The results were expressed as a percentage of viable cells in both non-induced and induced by oxidative stress cells. The results from three independence experiments of each four replicates were expressed as a percentage of the untreated control, which was assumed to have 100% viable cells.

### Proliferation of Lymphocytes

#### Ethic Statement

The animal experiments were approved by the Maejo University Animal Care and Use Committee (MACUC; Approval number MACUC 033S/2564), which is relevant to animal ethics for the use of animals for scientific purpose of the National Research Council of Thailand. The experiments were carried out on male Wistar rats (6 weeks of age; Wistar rats Hannover GALAS).

#### Isolation of Spleen Cells

Lymphocytes were produced from fresh entire rat spleens that had been treated as previously described (Cosenza-Contreras et al., 2019). The spleen (Wistar Hannover GALAS rats) was taken aseptically. The rat spleen was then placed on Petri plates with 1X Hank’s balanced salt solution (HBSS) buffer (Gibco, United States). Using a razor or scalpel blade, the spleen was carefully sliced into small pieces. Cell strainer (SPL, Korea) was placed over a conical tube with a capacity of 50 ml. The excised spleen was transferred twice into the cell strainer (70 μm) using a disposable transfer pipette. After that, the spleen cells were mashed or pressed through a sieve with the plunger end of a syringe. The spleen cells were washed through the cell strainer with RPMI-1640 (Gibco, United States) media. The spleen cells were suspended in RPMI-1640 media supplemented with 10% fetal bovine serum and 1% penicillin–streptomycin (10,000 U/ml penicillin and 10,000 μg/ml streptomycin; all from Gibco, United States) and adjusted to a concentration of 6 × 10^6^ cells/ml for further investigation.

#### Lymphocytes Stimulation Assay

The effects of PAAEs on lymphocyte proliferation were investigated using a modified MTT assay (Lee et al., 2011; Kumar et al., 2012). Mitogen-stimulated splenocyte proliferation was employed in the same way as lipopolysaccharide (LPS) and concanavalin A (ConA; both from Sigma, United States) used to stimulate B and T lymphocytes proliferation, respectively. Concentrations of PAAEs ranging from 0.02 to 0.2 mg/ml were conducted. Briefly, the spleen cell suspensions (100 μl) were added in a 96-well flat-bottom microtiter plate and then incubated with 50 μl of PAAEs. Consequently, the mitogen, either LPS or ConA, was applied to 50 μl/well at a final concentration of 10 μg/ml. The culture was incubated at 37°C, with 5% CO_2_, and 95% humidity for 48 h. Finally, lymphocytes proliferation was investigated by adding 10 μl of MTT for 4 h, after which the formazan crystals were dissolved with 100 μl of DMSO. The absorbance at 595 nm was measured by using a microplate reader. In following Eq. (7), lymphocyte proliferation was reported as a percentage. As a positive control, lymphocytes were stimulated in the presence of either LPS (10 μg/ml) or ConA (10 μg/ml).


(7) $$\mathrm{Lymphocyte proliferation rate}\;\left(\%\right)=\left[\frac{\mathrm{Abs}.\;\mathrm{of stimulated cells}}{\begin{array}{l} \mathrm{Abs}.\;\mathrm{of control}\;\left(non-\mathrm{stimulated}\right)\\ \;\mathrm{cells} \end{array}}\right]x\;100$$

### Statistical Analysis

All results were performed as mean with SD by Microsoft 365 Excel 2012. Moreover, these results were examined statistically through one-way ANOVA with SPSS statistical software (version 19.0). Duncan’s multiple range test evaluated the significance of the difference between groups based on a confidence level of 95.0%. Enzymatic hydrolysis condition was analyzed using Design-Expert program version 11 from Stat-Ease, Inc., Minneapolis, United States. The IC_50_ values were performed with the GraphPad PRISM 9 program (GraphPad Software, Inc., San Diego, CA, United States).

## Results

### Earthworm Identification

The sequence was determined and contig assembly program the sequence using BioEdit version 7.0.4. The nucleotide was run through BioEdit, which was blasted on NCBI database. As the result, earthworm was identified as *A. arenulus* (accession no. KU565182.1). This earthworm *A. arenulus* was revealed to be a member of the genus *Amynthas* of the family Megascolecidae. COI gene sequence exhibited 86.13% similarity with species according to the NCBI database.

### Proportion of Earthworm Protein Isolates

The isolated proteins were found to be 14.02 ± 0.17%, which the protein isolates were extracted from defatted sample by isoelectric precipitation. Isolated protein analysis using the Kjeldahl technique found 55.39 ± 2.30%.

### Characterization of Protease-Producing Bacteria and Protease

Fifty-eight bacteria isolates were obtained from water using the earthworm carcass (48 isolates) and dried earthworm (10 isolates) soaking processes. After screening, 54 isolates were found to produce protease (Table 3). The PM 35 had the highest protease production, with protease-producing zone, protein content, and protease activity of 38.33 mm, 896.55 μg/ml, and 6.13 U/ml, respectively. The crude protease had a specific activity of 6.83 U/mg, whereas purified protease had a specific activity of 19.54 U/mg as shown in Table 4.

**Table 3:** The protease-producing bacterial activity with clear zone and protein contents.

Isolates	Inhibition zone (mm)	Protein content (μg/ml)	Isolates	Inhibition zone (mm)	Protein content (μg/ml)
PM 1	27.18 ± 0.29	644.83 ± 1.24	PM 30	37.00 ± 0.00	116.09 ± 9.16
PM 2	22.10 ± 0.84	583.91 ± 2.45	PM 31	38.67 ± 2.13	126.44 ± 5.52
PM 3	20.88 ± 0.85	579.31 ± 41.95	PM 32	-	-
PM 4	22.00 ± 0.00	94.25 ± 10.72	PM 33	37.23 ± 0.40	637.93 ± 6.29
PM 5	22.13 ± 0.87	408.05 ± 8.55	PM 34	38.23 ± 0.40	742.53 ± 7.17
PM 6	-	-	PM 35	38.33 ± 0.58	896.55 ± 4.83
PM 7	18.60 ± 0.71	120.69 ± 7.24	PM 36	38.87 ± 1.03	709.20 ± 16.61
PM 8	20.50 ± 0.58	106.90 ± 9.86	PM 37	40.83 ± 1.61	724.14 ± 8.06
PM 9	21.94 ± 0.47	162.07 ± 23.36	PM 38	-	-
PM 10	37.83 ± 0.91	677.01 ± 9.66	PM 39	35.00 ± 1.00	673.56 ± 9.20
PM 11	38.00 ± 1.00	618.39 ± 23.46	PM 40	26.85 ± 3.75	665.52 ± 11.06
PM 12	34.67 ± 0.58	642.53 ± 11.74	PM 41	26.33 ± 3.79	491.95 ± 2.45
PM 13	35.33 ± 0.58	652.87 ± 12.23	PM 42	31.33 ± 0.58	688.51 ± 6.32
PM 14	36.67 ± 1.15	654.02 ± 3.13	PM 43	40.00 ± 0.00	524.14 ± 9.66
PM 15	38.12 ± 1.53	547.13 ± 12.23	PM 44	34.52 ± 2.54	535.63 ± 15.13
PM16	33.67 ± 2.08	616.09 ± 3.13	PM 45	34.87 ± 0.23	621.84 ± 8.62
PM 17	38.53 ± 4.80	618.39 ± 4.39	PM 46	33.33 ± 2.08	582.76 ± 5.89
PM 18	38.67 ± 1.15	613.79 ± 9.19	PM 47	34.67 ± 2.31	632.18 ± 9.56
PM 19	34.87 ± 1.80	663.22 ± 6.67	PM 48	33.68 ± 2.25	693.10 ± 3.98
PM 20	37.33 ± 0.58	611.49 ± 7.96	EDNP 1	28.80 ± 0.72	405.75 ± 16.27
PM 21	34.88 ± 0.20	706.90 ± 9.87	EDNP 2	28.43 ± 0.51	672.41 ± 3.06
PM 22	38.00 ± 0.00	648.28 ± 1.24	EDNP 3	28.00 ± 1.73	604.60 ± 2.02
PM 23	36.67 ± 0.58	631.03 ± 3.83	EDNP 4	27.60 ± 1.04	626.44 ± 7.77
PM 24	33.33 ± 1.53	620.69 ± 6.33	EDNP 5	27.67 ± 0.58	693.10 ± 14.05
PM 25	37.00 ± 1.00	479.31 ± 2.15	EDNP 6	25.00 ± 0.60	693.10 ± 3.29
PM 26	31.02 ± 2.61	167.82 ± 7.93	EDNP 7	24.67 ± 0.58	725.29 ± 2.11
PM 27	38.00 ± 1.00	314.94 ± 6.29	EDNP 8	27.60 ± 0.36	774.71 ± 6.13
PM 28	37.20 ± 2.71	178.16 ± 3.78	EDNP 9	29.33 ± 0.58	824.14 ± 7.93
PM 29	-	-	EDNP 10	28.40 ± 1.85	748.28 ± 14.34

The mean with SD of three replications was used to represent the data.

**Table 4:** *Bacillus velezensis* PM 35 exhibited the specific activity of crude protease and purified protease.

Purification steps	Total activity (U/ml)	Total protein (mg)	Specific activity (U/mg)	Weight of protein (g)	Yield (%)
Crude protease	6.13 ± 0.231	0.89 ± 0.048	6.83 ± 0.009	nd.	nd.
Acetone precipitation (before lyophilized)	nd.	nd.	nd.	60	100
Acetone precipitation (after lyophilized)	14.38 ± 0.028	0.73 ± 0.029	19.54 ± 0.004	0.3	0.5

The mean with SD of three replications was used to represent the data (nd, no determined).

### Morphology and Identification of Protease-Producing Bacteria

The colony morphology of the PM 35 isolate was white, irregular, and round with an undulating margin. Isolate PM 35 was Gram-positive bacteria, rod-shaped, had a central endospore position, and produced catalase. This bacterium was identified using 16S rRNA and compared to the database, which showed that it was related to *Bacillus* sp. The first pair of primers could not identify the bacterial species. Thus, to confirm the species of *Bacillus*, the second pair of primers, 16S–23S rRNA internal transcribed spacers, was used. Bacterium was found to be 99.22% identical to *Bacillus velezensis* (accession no. MW136663.1).

### Yields of Protein Hydrolysate

The E/S concentration ratio and hydrolysis time ranges were optimized using the RSM for maximal protein hydrolysate. The influence of protease hydrolysis factors on the protein hydrolysates was investigated by ANOVA. The value of *p*, less than 0.05, indicated a significant effect on protein hydrolysate yields.

The experimental data of the model that was supposed to fit were proved by the lack of fit test to be not significant (value of *p* > 0.05). Both the E/S concentration ratio and time influenced the protein hydrolysate with a regression coefficient (*R*^2^ = 0.9547). Following coded Eq. (8) represents the relationship between protein hydrolysate yields and the hydrolysis variable.


(8) $$\begin{array}{l} \mathrm{Yields}\;\mathrm{of}\;\mathrm{protein}\\ \;\mathrm{hydrolysates}\;\left(\begin{array}{l} g/g\;\\ \mathrm{protein} \end{array}\right)=10.76+1.17A+3.50B+\\ \;2.59\mathrm{AB}+3.10A^{2}+3.16B^{2} \end{array}$$

**Figure 1:**
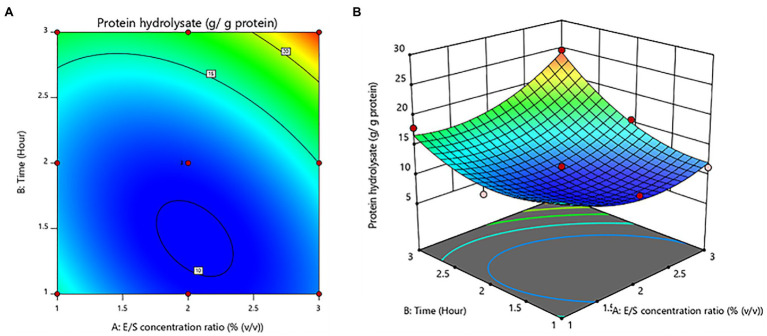
Contour plot **(A)** and response surface plot **(B)** yield of protein hydrolysates as a function of enzyme/substrate concentration ratio and time.

Furthermore, time was shown to be a significant (value of *p* < 0.05) variable influencing protein hydrolysate yields. The E/S concentration ratio was not statistically significant (value of *p* > 0.05). For protein hydrolysate yields, there was a notable interaction effect between E/S concentration ratio and time. The contour plot and response surface plot of the E/S concentration ratio and time on the yield of protein hydrolysates are shown in Figure 1. The yield of protein hydrolysates increased when the hydrolysis time was enhanced. While the E/S concentration ratio increased, the yield of protein hydrolysates did not increase significantly. Thus, the maximum yield of protein hydrolysates predicted by RSM design was 24.62 ± 0.09 g/g protein with an optimal E/S concentration ratio of 3% (v/v) and a hydrolysis time of 3 h.

### Degree of Hydrolysis of Protein Hydrolysate From *Amynthas arenulus* Earthworm

The suitable ranges of E/S concentration ratio and time for DH of protein hydrolysates were evaluated by ANOVA to determine the influence of these hydrolysis factors on the DH. The model was significantly showed a lack of fit (value of *p* < 0.05). The variables of hydrolysis have a quadratic association with a regression coefficient (*R*^2^ = 0.8502). Coded Eq. (9) shows the association.


(9) $$\begin{array}{l} \mathrm{Degree}\;\mathrm{of}\;\mathrm{hydrolysis}=10.99+9.88A+12.22B+\\ \;4.68\mathrm{AB}+27.10A^{2}+9.82B^{2} \end{array}$$

**Figure 2:**
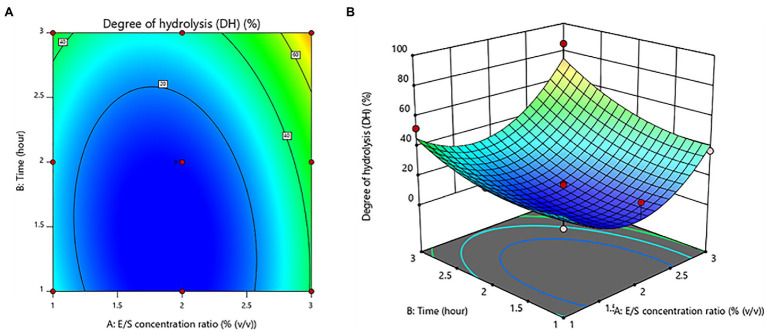
Contour plot **(A)** and response surface plot **(B)** of the degree of hydrolysis (DH) as a function of enzyme/substrate concentration ratio and time.

The E/S concentration ratio and time had no significant (value of *p* > 0.05) effects. On the other hand, the E/S concentration ratio was shown to be a significant quadratic component (value of *p* < 0.05). Figure 2 depicts the contour plot and response surface plot of the E/S concentration ratio and time on the DH percentage of protein hydrolysates. Since the hydrolysis of the E/S concentration ratio increased from 1% to 3% (v/v), the DH percentage has increased as well. Similarly, increasing hydrolysis time was obvious to produce a higher % DH. As a result, the optimum hydrolysis conditions were predicted to be a 3% (v/v) E/S concentration ratio and a time of 3 h for the highest DH of 85.45 ± 0.69%.

### Gastrointestinal-Resistant Protein Hydrolysates and Fractionation

**Figure 3:**
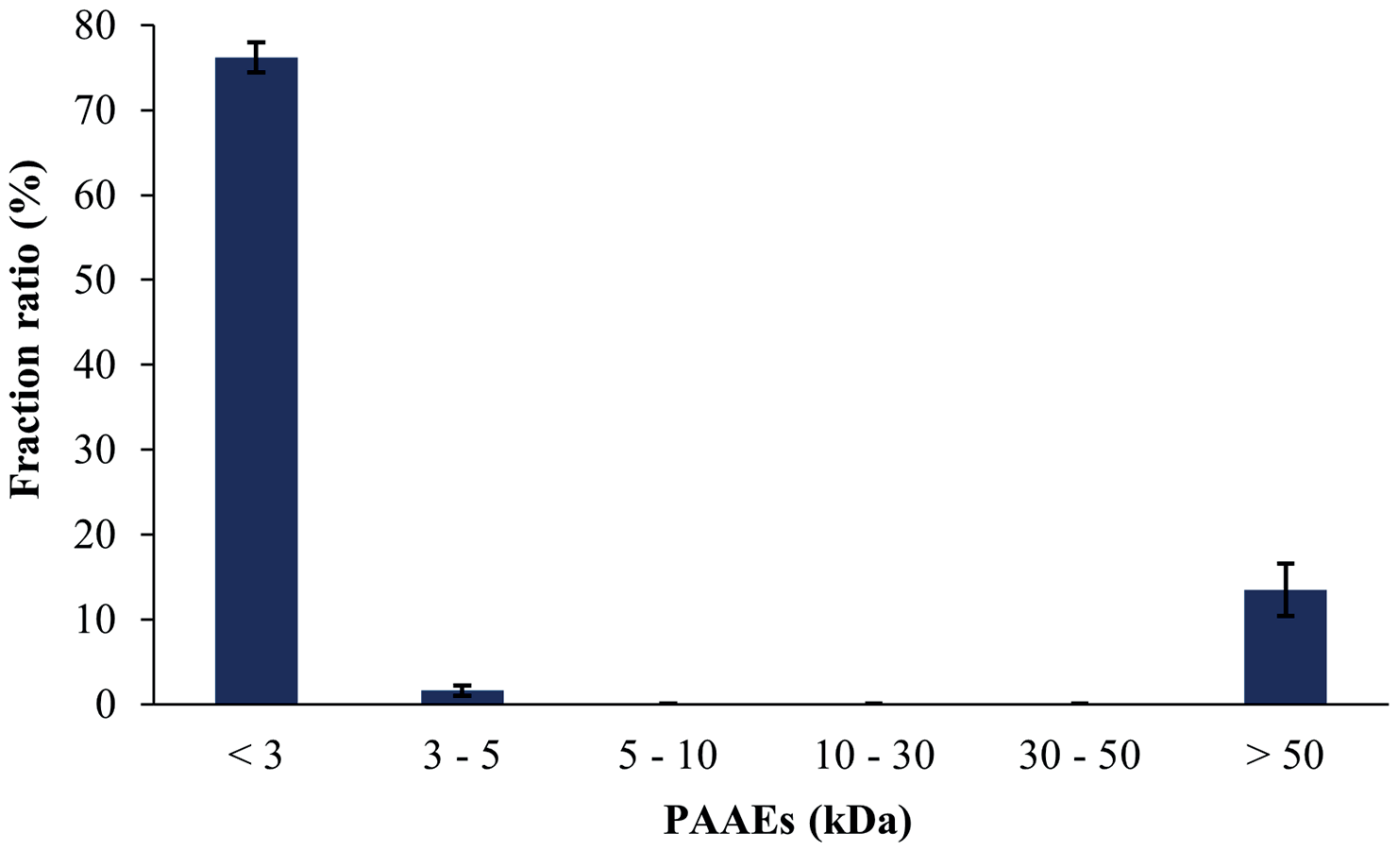
The fraction ratios of gastrointestinal environment-resistant earthworm peptides were permeated through the molecular weight cutoffs (MWCO) of 50, 30, 10, 5, and 3 kDa membranes. The results were expressed as mean with SD of triplicates from three independent studies.

The peptides resisted on the gastric and intestinal environments, which were found to be 25.95% and 30.93%, respectively (Table 5). The peptides from the *A. arenulus* earthworm (PAAEs) were permeated through the 50, 30, 10, 5, and 3 kDa membranes, which showed 6 MWCO fractions. The fraction ratio of PAAEs was found to be MWCO <3, 3–5, 5–10, 10–30, 30–50, and >50 kDa fractions, which presented 76.21%, 1.60%, 0.08%, 0.05%, 0.04%, and 13.48%, respectively (Figure 3).

**Table 5:** The gastrointestinal environment-resistant earthworm peptides.

Initial protein hydrolysates (g)	Gastric-resistant environment (g)	Intestinal-resistant environment (g)	Peptides (%)
5	1.30 ± 0.10	nd.	25.95
2.5	nd.	0.77 ± 0.03	30.93

The results are generated in triplicate (nd, not determined).

### Proliferative Inhibition of Liver Cancer Cells

**Figure 4:**
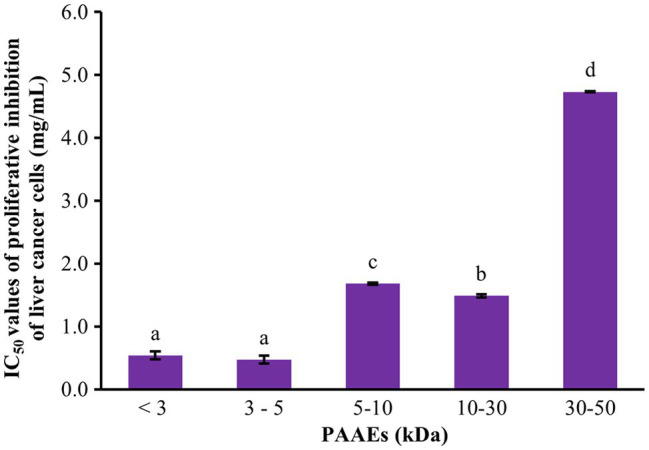
Cytotoxic effect of PAAEs on proliferative inhibition of HepG2 cells. The results were expressed as mean with SD of three replications from three independent studies. ^a–d^The values with different letters appearing show significant differences (*p* < 0.05).

The cytotoxic effect of PAAEs against liver cancer was investigated. The MWCO 5 fractions of PAAEs inhibited cell proliferation, which was discovered to be MW <3, 3–5, 5–10, 10–30, and 30–50 kDa. Figure 4 shows the cytotoxic effects of the IC_50_ values of PAAEs on HepG2 cells. The IC_50_ values for the MW <3, 3–5, 5–10, 10–30, and 30–50 kDa fractions were 0.54, 0.47, 1.68, 1.49, and 4.73 mg/ml, respectively. While comparing the PAAEs fractions, the MW < 3 kDa and MW 3–5 kDa were more effective at inhibiting the growth of HepG2 cells than the larger MWCO fractions, which was significant (*p* < 0.05). As a result, the IC_50_ values of MW <3 and 3–5 kDa were chosen for further investigation.

### Cytotoxicity of Normal Mouse Fibroblast Cells

**Figure 5:**
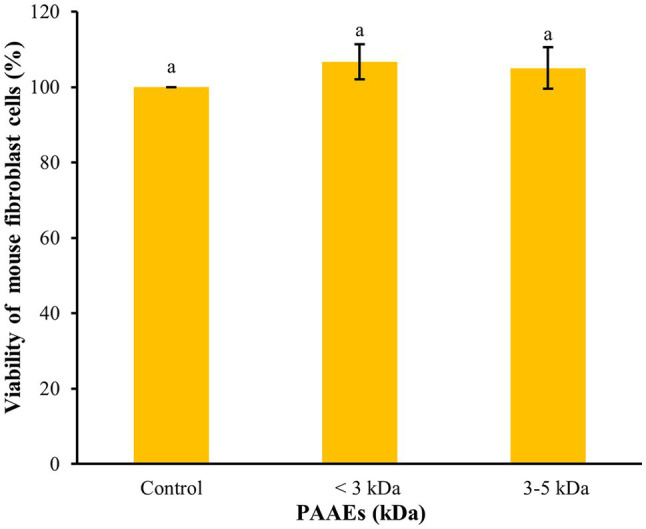
The activity of PAAEs (MW < 3 and 3–5 kDa, IC_50_ values) on cytotoxic to normal mouse fibroblast cells (L929). The results were expressed as mean with SD of triplicates from three independent studies. ^a^The values with different letters appearing show significant differences (*p* > 0.05).

The MTT assay was used to assess the cytotoxicity of PAAEs in L929 cells in terms of cellular viability. Figure 5 shows that the PAAEs MW <3 and 3–5 kDa fractions demonstrated non-cytotoxicity toward L929 cells under IC_50_ concentration of 0.54, 0.47 mg/ml. The IC_50_ values of PAAEs MW <3 and 3–5 kDa fractions were not toxic to normal mouse fibroblast cells, with survival rates of 106.69% and 105.04%, respectively, as compared to the control (100%).

### Morphological Changes and Apoptosis of Liver Cancer Cells

#### Morphological Changes in HepG2 Cells

**Figure 6:**
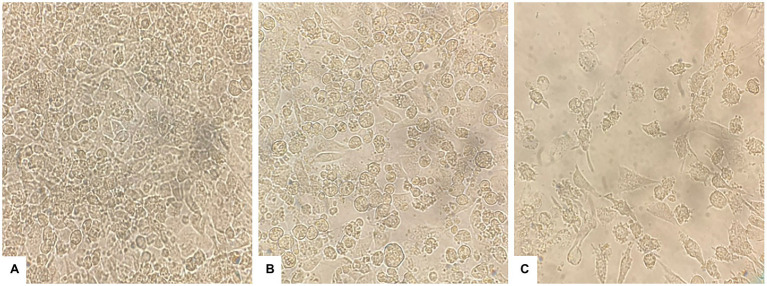
PAAEs triggered morphological and apoptotic changes in HepG2 cells. These cell morphological changes were observed using an inverted microscope (original magnification, x400). **(A)** Control (untreated cells), **(B)** cells treated with 0.5% (v/v) dimethyl sulfoxide (DMSO) as comparative group, and **(C)** cells treated with PAAEs (MW <3 kDa, IC_50_ value at 0.54 mg/ml).

The effect of PAAEs treatment revealed morphological changes in HepG2 cells. The control cells showed no changes due to their high density, a normal phenomenon of cultured cells (Figure 6A). Meanwhile, when exposed to 0.5% (v/v) DMSO and PAAEs MW < 3 kDa (0.54 mg/ml) for 24 h (Figures 6B,C), HepG2 cells demonstrated differing degrees of morphological changes, including loss of cell adhesion, cell shrinkage, membrane blebbing, cytoplasm enlargement, cell fragmentation, and the formation of apoptotic bodies, which eventually resulted in lysis of these bodies.

#### Visualization of Morphological Apoptotic Changes by Dual AO/PI Staining

**Figure 7:**
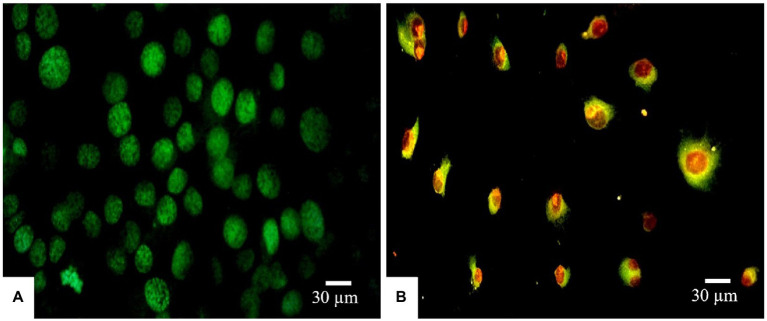
Induced apoptosis progression in HepG2 cells treated with PAAEs. Morphology of cells was subjected in fluorescent microscopy (original magnification, x400). Under the fluorescent microscope, HepG2 cells exhibited apoptotic effects which treated with PAAEs (MW <3 kDa, IC_50_ value at 0.54 mg/ml). **(A)** Untreated cells were stained green. **(B)** Apoptotic cells were triggered by the PAAEs.

Morphological apoptotic changes were assessed using AO/PI fluorescence microscopy double staining after treatment for 24 h. The IC_50_ values of PAAEs MW < 3 kDa (0.54 mg/ml) enhanced apoptotic morphological changes in HepG2 cells. The cells were labeled with AO/PI interpolating nucleic acid dyes, which bind to DNA and emit green and orange fluorescence. The control (untreated cells) exhibited brilliant green, indicating that they were live cells (Figure 7A). Meanwhile, cells in late apoptosis have a compact nuclear structure and a reddish-orange color. Cells treated with PAAEs showed typical morphological characteristics of apoptotic cell death, including cell shrinkage or convolution, nucleus fragmentation, chromatin condensation, membrane blebbing, membrane swelling, and apoptotic bodies (Figure 7B).

### DNA Fragmentation

**Figure 8:**
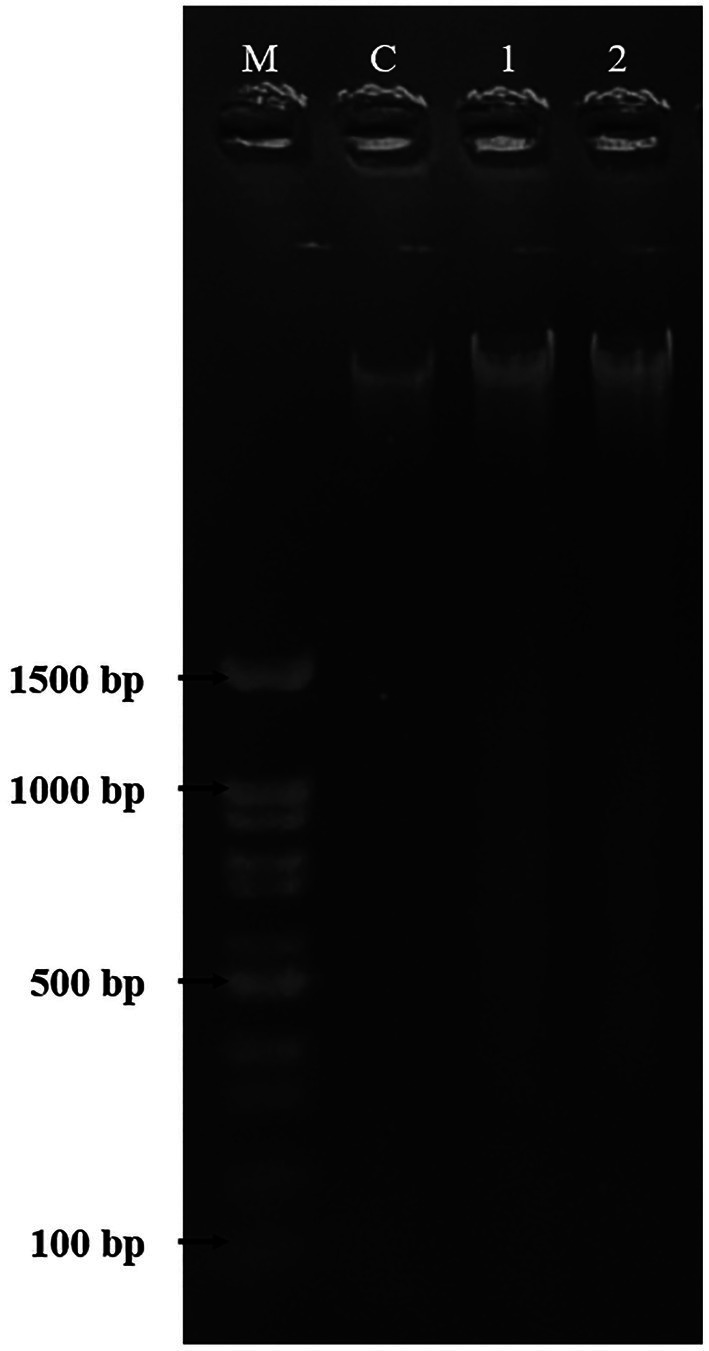
The HepG2 cells treated with DMSO (0.5%) as comparative group and PAAEs (MW <3 kDa, IC_50_ value at 0.54 mg/ml). DNA fragmentations were visualized under UV light with ethidium bromide staining. Lane M, DNA marker; Lane C, untreated cells; Lane 1, cells treated with 0.5% DMSO; and Lane 2, cells treated with PAAEs.

DNA fragmentation was used to confirm and observe the occurrence of apoptosis in HepG2 cells. As shown in Figure 8, the HepG2 cells were untreated and treated with DMSO [0.5% (v/v)] and PAAEs MW < 3 kDa (0.54 mg/ml). Non-fragmented DNA was presented in the control group (untreated cells) of liver cancer cells for 24 h (land C). Small and smaller DNA fragments of liver cancer cells treated with 0.5% (v/v) of DMSO (land 1) and 0.54 mg/ml of PAAEs MW < 3 kDa (land 2) were, respectively, noticed.

### Antioxidant Activity

**Figure 9:**
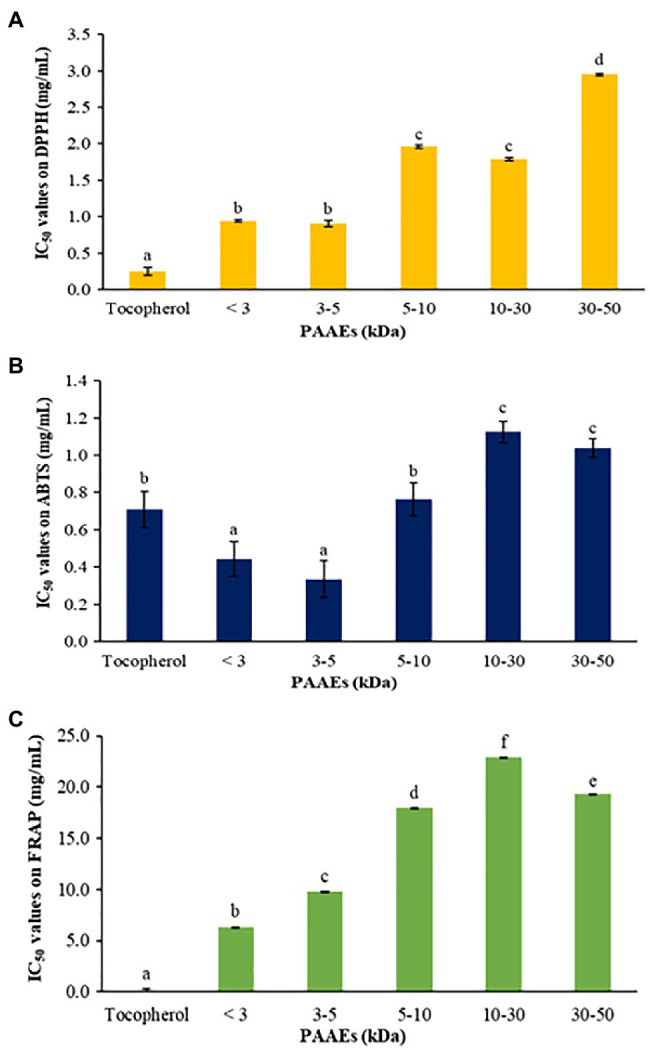
The PAAEs activities against DPPH radical scavenging activity **(A)**, ABTS radical scavenging activity **(B)**, and ferric reducing antioxidant powder **(C)**. The results were expressed as mean with SD of three replications. ^a–f^The values with different letters show significant differences (*p* < 0.05).

The antioxidant abilities of PAAEs were assessed by DPPH radical scavenging activity, ABTS radical scavenging activity, and the FRAP assay. The IC_50_ values of PAAEs (MW <3, 3–5, 5–10, 10–30, and 30–50 kDa fractions) against DPPH are summarized in Figure 9A. The IC_50_ values for MW < 3 kDa were 0.94 mg/ml, 0.91 mg/ml for MW 3–5 kDa, 1.96 mg/ml for MW 5–10 kDa, 1.79 mg/ml for MW 10–30 kDa, and 2.95 mg/ml for MW 30–50 kDa, respectively.

Meanwhile, the PAAEs against ABTS are summarized in Figure 9B. The IC_50_ values of PAAEs of MW < 3 kDa (0.44 mg/ml) and 3–5 kDa (0.33 mg/ml) were higher than those of MW 5–10 kDa (0.76 mg/ml), MW 10–30 kDa (1.12 mg/ml), and MW 30-50 kDa (1.04 mg/ml), respectively.

For the PAAEs against FRAP are summarized in Figure 9C. The IC_50_ values of the PAAEs MW <3, 3–5, 5–10, 10–30, and 30–50 kDa fractions were found to be 6.34, 9.80, 17.99, 22.92, and 19.27 mg/ml, respectively.

### Protective Effect of PAAEs on the Viability of Oxidation-Induced Cells

**Figure 10:**
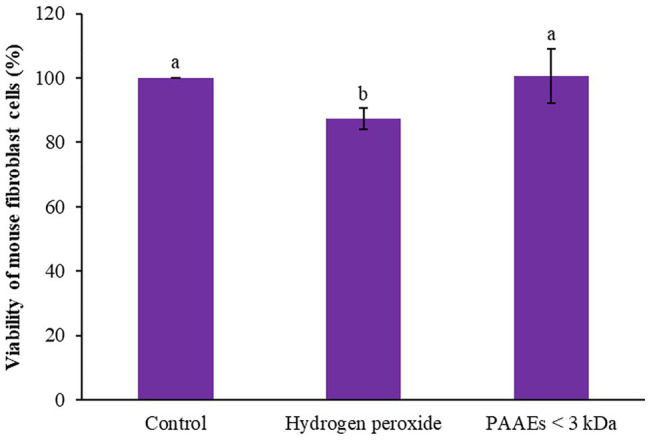
Effect of PAAEs (MW <3 kDa, IC_50_ value at 0.54 mg/ml) on the protective activity against oxidative stress-induced cells. The results were expressed as mean with SD of three replications from three independent studies. The letters above the bars denote significant difference (*p* < 0.05).

The capability of PAAEs MW < 3 kDa (0.54 mg/ml) on mouse fibroblast cells induced by oxidative stress was investigated. Hydroxide peroxide (final conc. 50 μM) was then administered to the cells, which showed an 87.35% of survival rate when compared to untreated cells (100%), which was significant (*p* < 0.05). Interestingly, the PAAEs MW < 3 kDa safeguarded the cells from oxidative stress by 100.65%, which was significantly higher (*p* < 0.05) than the comparative H_2_O_2_ group as shown in Figure 10.

### Induction of Lymphocyte Proliferation

**Figure 11:**
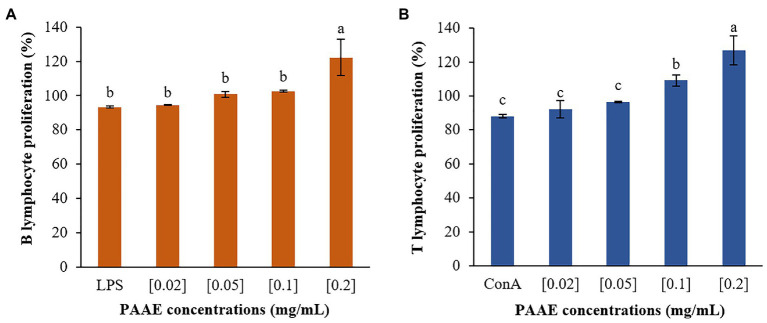
The high-potential PAAEs stimulated B lymphocytes **(A)** and T lymphocytes **(B)** proliferation. The lymphocytes were derived from Wistar Hannover GALAS rats’ spleen cells. Positive controls include lipopolysaccharide (LPS) and concanavalin A (ConA). The results were expressed as mean with SD of three replications. The letters above the bars represent a statistically significant difference (*p* < 0.05).

Researchers discovered that PAAEs MW < 3 kDa stimulated lymphocyte proliferation and had high potential. The 0.2 mg/ml concentration of PAAEs was shown to significantly proliferate B lymphocytes by 122.25% (*p* < 0.01) when compared to LPS (93.38%). Meanwhile, B lymphocytes were stimulated with PAAEs at 0.02, 0.05, and 0.1 mg/ml, achieving 94.41%, 100.77%, and 102.59%, which was not significant (*p* > 0.05) when compared to LPS (Figure 11A).

Furthermore, PAAEs (0.2 mg/ml) were capable of proliferating T lymphocytes by 126.71%, which was significant (*p* < 0.05). When compared with ConA (88.07%), the PAAEs at 0.02, 0.05, and 0.1 mg/ml concentrations were capable of stimulating T lymphocytes by 92.20%, 96.60%, and 109.24% (*p* > 0.05), respectively (Figure 11B).

## Discussion

Earthworms have been used as a new alternative protein source. Traditional medicine has made extensive use of earthworms. Its medical efficacy is related to its bioactive compounds in the body. Because of the high protein content of earthworms, they are usually presented. Researchers discovered an extraordinarily high protein content (55.39%) in *A. arenulus* earthworm, which is exclusively found in the northeast of Thailand (Bantaowong et al., 2014). Research had shown the potential utility of earthworm as protein sources. Similarly, previous research on the Megascolecidae family revealed protein content of *Perionyx excavates* earthworm (44.3%; Pucher et al., 2014) and *Eudrilus eugeniae* earthworm (29.49%; Deepika et al., 2018). Earthworm powder is highly recommended as a high-protein source. Researchers have proposed that earthworm powder is used as a nutritional supplement not only in the diets of fish and other animals but also in humans. These results demonstrated that earthworm is high in protein and encourages its prospective use as a nutritious food supplement.

Novel alternative protein hydrolysates or bioactive peptides have increasingly been used in therapeutic strategies. Because a novel protein hydrolysate can be created from protein sources and hydrolyzed enzyme, other optimum variables are also considered, such as pH, incubation time, and temperature (Udenigwe and Aluko, 2012). In this work, we discovered a high-potential, new *B. velezensis* PM 35 capable of hydrolyzing earthworm proteins. Many proteins can be limitedly hydrolyzed, yielding peptides, also known as protein hydrolysates, with extraordinary biological activity such as antihypertensive, immune-stimulating, antimicrobial, and antioxidant activities (Danquah and Agyei, 2012).

As new protease-producing *B. velezensis* PM 35 was characterized in this study. The genus *Bacillus* is probably the most important bacterial source of proteases and is capable of producing high yields of neutral and alkaline proteolytic enzymes. *Bacillus* proteases are predominantly alkaline and have pH activity values above 7.0, which means have an optimal pH range between 6 and 10, with optimal temperatures between 35°C and 60°C (Anbu, 2013). *Bacillus* proteases have several remarkable characteristics for many industrial applications. Because of their broad pH and temperature activity and stability range, *Bacillus* proteases have been extensively purified and characterized regarding their properties for use in the food industry, pharmaceutical industry, and biotechnological applications (Annamalai et al., 2013). Similarly, the optimal conditions of extracellular protease-producing bacteria were concerned with *B. velezensis* (Khalid et al., 2021). The *B. velezensis* bacteria were found 19.54 U/mg of enzyme activity, which is similar to *B. pumilus* (63.58 U/ml; Gomaa, 2013) and *B. subtilis* (27.19–60.50 U/ml; Rodarte et al., 2011; Rahman et al., 2018). As a result, proteolytic enzymes generated from *Bacillus* proteases have been shown to produce intriguing outcomes.

A central composite design of response surface methodology was applied to optimize hydrolyzing *A. arenulus* earthworm’s proteins, specifically, the E/S concentration ratio [1%–3% (v/v)] and time (1–3 h). Researchers discovered the new protein hydrolysates (24.62%), which were shown to have a high DH of 85.45%. There was no report that used protease-producing bacteria to hydrolyze *A. arenulus* earthworm’s proteins. Likewise, the protein hydrolysates from oysters were performed with 0.4% of protease for 6 h, which showed 27.97% of DH (Li et al., 2019). Hydrolyzing cuttlefish proteins were carried out using 3 U/mg of E/S for 4 h, and the result was discovered to be 15% of DH (Balti et al., 2011). These findings demonstrate that hydrolyzing protein with protease enzyme was performed under controlled conditions and in a short period of time, demonstrating a valid method for extracting high-quality protein from this earthworm to produce suitable ingredients for food and medicinal purposes.

The researchers discovered new molecular weights of less than 3, 3–5, 5–10, 10–30, 30–50, and greater than 50 kDa in PAAEs. The PAAEs MW < 3 kDa had the greatest fraction ratio of 76.32%. Protein hydrolysis in the human body occurs during gastric and pancreatic digestion, followed by absorption in the intestines. Peptides are absorbed in the form of free amino acids or oligopeptides. Generally, peptides with a lower molecular weight (<10 kDa) were accessible and hence had a high potential for influencing the functioning of the human body (Li et al., 2019). These results reveal that under suitable conditions, utilizing new *B. velezensis* PM 35, the greatest PAAEs MW < 3 kDa could be obtained.

The novel PAAEs (MW < 3 kDa) dramatically inhibited the proliferation of HepG2 cells, which presented an IC_50_ value of 0.54 mg/ml. Likewise, mucus proteins extracted from *E. eugeniae* earthworm and *Perionyx excavatus* earthworm were found to be 0.14 mg/ml (MW 8–60 kDa) and 0.17 mg/ml (MW 12–100 kDa), respectively, which inhibited HepG2 cells (Prem-U-Domkit et al., 2017). The coelomic fluid protein from *Pheretima posthuman* earthworm prevented MCF-7 cells growth, with an IC_50_ value of 0.26 mg/ml (MW 15 kDa; Verma and Sobha, 2013). The low molecular weight of gastrointestinal-resistant protein hydrolysates appeared to improve interactions with cancer cell components and thus enhance anticancer activity (Kim, 2011). Importantly, the cancer cell membrane is a key role in the selectivity of anticancer peptides (Quemé-Peña et al., 2021). As on the outer leaflet of healthy cell membranes are mostly comprising of neutral zwitterionic phospholipids, such as phosphatidylcholine and sphingomyelin (Hoskin and Ramamoorthy, 2008; Chiangjong et al., 2020). In contrast, cancer cell membranes normally carry a net negative charge of phosphatidylserine which contains 3%–9% of the total membrane phospholipids (Riedl et al., 2011). The postulated mechanisms or action modes of anticancer peptide are comprising of pore formation in the lipid (barrel-stave and toroidal pore models), the thinning of the membrane bilayer, membrane dissolution (carpet model), or lipid-peptide domain formation (Oelkrug et al., 2015; Wang et al., 2017). Of our results, the low molecular weight peptides may correlate with these mechanisms higher than the large ones, thus achieving better anticancer potential. Moreover, the new PAAEs (MW < 3 kDa) were discovered to be non-cytotoxic on normal mouse fibroblast cells. These results suggested that the PAAEs with MW approximately <3 kDa and 3–5 kDa could be applied in prophylactic strategy for liver cancer.

New PAAEs (MW < 3 kDa) triggered morphological apoptosis in HepG2 cells. Cell shrinkage, nucleus fragmentation, chromatin condensation, membrane blebbing, and membrane swelling have been described as characteristics of apoptotic cell death inducing (Amini-Sarteshnizi et al., 2014) and were observed in our research. Apoptosis was also investigated in relation to DNA fragmentation. As a result, small DNA fragments were seen in HepG2 cells treated for 24 h with the novel PAAEs (MW < 3 kDa). According to our findings, novel PAAEs (MM < 3 kDa) induced apoptosis in liver cancer cells. Similar work was published in which the protein-carbohydrate extracts from *Dendrobaena veneta* earthworm induced apoptosis in HT-29 cancer cells by activating procaspase-3 (Czerwonka et al., 2020). The fibrinolytic enzyme of *Eisenia foetida* earthworm could trigger apoptosis in hepatoma cells (HLE and Huh7), revealing the appearance of green areas of broken and condensed chromatin (Chen et al., 2007). These our results showed similar report that the *A. arenulus* peptides could induce cancer cell death *via* an apoptotic mechanism because of their low molecular weight (Chi et al., 2015).

An antioxidant protects against an overabundance of reactive oxygen species (ROS). Excess ROS levels cause oxidative stress-related diseases such as cancer, diabetes, atherosclerosis, and arthritis (Borges and Castle, 2015; Wattanasiritham et al., 2016). In this research, the new PAAEs with MW < 3 kDa were examined against the extremely high antioxidant activity, which was exhibited on DPPH, ABTS, and FRAP. The antioxidant peptides passed through the gastrointestinal tract and revealed powerful residues within their amino acid sequences, resulting in the peptide’s great antioxidant activity (Udenigwe and Aluko, 2012). However, the antioxidant activity of peptides is dependent on their molecular weight, with smaller molecular weight peptides being easier to absorb into the intestinal epithelium, suggesting that they often have beneficial biological functions (Wang and De Mejia, 2005). It has also been postulated that the low molecular weight of gastrointestinal-resistant peptides, the greater the probability it has of penetrating the intestinal barrier and exerting a biological effect (Qian et al., 2008). Similarly, antioxidant peptides of clam worms exhibited DPPH activity (IC_50_ of 0.017 mg/ml; Park et al., 2020). Furthermore, the antioxidant activity of *Lumbricus rubellus* earthworm exhibited a DPPH with IC_50_ of 12.33 mg/ml (Dewi et al., 2017). These findings reveal that the PAAEs (MW < 3 kDa) of *A. arenulus* earthworm were capable of antioxidant capacity.

The production of ROS, as a significant biomarker for assessing the state of oxidative stress in cells, could not only operate as hazardous molecules but also play a variety of critical roles as signaling molecules controlling many biological processes. Hydrogen peroxide has been researched as an oxidant reagent that is hazardous to cells, such as fibroblasts, because of the presence of an unstable and highly reactive molecule, such as •OH. As a result, excessive ROS generation could result in cell death. We discovered that the novel PAAEs (MW < 3 kDa) exhibited a high efficiency in protecting normal mouse fibroblast cells from oxidative stress, higher than cells treated with hydrogen peroxide. Similarly, extracts of *Koelreuteria henryi* could reduce H_2_O_2_-induced cellular oxidative damage in L929 cells by 71.4%–98.9% (Oliveira et al., 2019). These studies suggest that new PAAEs (MW < 3 kDa) are capable of protective activity against oxidative stress, which is the primary cause of human diseases like cancer.

Stimulated lymphocyte proliferation is generally triggered by mitogens such as LPS and ConA. In this research, proliferative lymphocytes, derived from freshly isolated murine splenocytes, were stimulated with the novel PAAEs (MW < 3 kDa), LPS, and ConA. The novel PAAEs stimulated proliferation of B lymphocytes (122.25%) and T lymphocytes (126.71%) higher than LPS (93.38%) and ConA (88.07%), respectively. Interestingly, this effective dose was only 0.2 mg/ml which lowers than that of the IC_50_ concentration of proliferative inhibition of liver cancer cells at 0.54 mg/ml, and this concentration may respond to individual cells of different body organ or system. Similarly, effects of oyster hydrolysates increased T lymphocyte proliferation as 104.18% (0.25 mg/g), 120.87% (0.5 mg/g), and 127.06% (1 mg/g; Wang et al., 2010). However, there have been few reports on the immunomodulatory activity of earthworm peptides produced using enzyme technology. Earthworm peptides, as dietary proteins, are anticipated to include a significant source of biological peptides, which could serve as a potential resource for immunomodulatory.

## Conclusion

Based on these results, our findings indicated that the low molecular weight of bioactive peptide as PAAE (MW < 3 kDa) produced by protease recovered from novel *B. velezensis* PM 35 prevented the proliferation of HepG2 liver cancer cells through an apoptotic inducing cell death program. They also had antioxidant properties, could protect against oxidative stress-induced cells, and induced immune lymphocyte proliferation. Therefore, this bioactive earthworm peptide could be considered as a potential biotherapeutic agent for human diseases, especially for liver cancer bioprophylactic and/or therapy. The bioactive peptide can be further utilized in the broad applications, especially in the food and medicine industries. Our future researches will focus on the mechanisms of anticancer peptide on cell apoptosis in other cancers and anti-inflammation activity. Furthermore, this beneficial *B. velezensis* PM 35 strain will further investigate in various biotechnological and medical applications.

## Data Availability Statement

The original contributions presented in the study are included in the article/supplementary material, further inquiries can be directed to the corresponding author.

## Ethics Statement

The animal study was reviewed and approved by the Maejo University Animal Care and Use Committee (MACUC; Approval number MACUC 033S/2564), which is relevant to animal ethics for the use of animals for scientific purpose of the National Research Council of Thailand.

## Author Contributions

MT contributed to conceptualization, designed and conceived the experiments, funding acquisition, resources, supervision, investigation, final revision, and editing. PW conducted the experiments, wrote and prepared the draft, and did the data analysis, review, and editing. All authors contributed to revising the manuscript, reading and approving the final version, and arranging the database.

## Funding

This work is supported by the Research and Researcher for Industries Ph.D. program (RRi: PHD60I0065) of the National Research Council of Thailand (NRCT), under Thailand Science Research and Innovation (TSRI), in recognition of the financial assistance offered which enabled this research study to be completed.

## Conflict of Interest

The authors declare that the research was conducted in the absence of any commercial or financial relationships that could be construed as a potential conflict of interest.

## Publisher’s Note

All claims expressed in this article are solely those of the authors and do not necessarily represent those of their affiliated organizations, or those of the publisher, the editors and the reviewers. Any product that may be evaluated in this article, or claim that may be made by its manufacturer, is not guaranteed or endorsed by the publisher.

## Footnotes

^1^ http://www.ncbi.nlm.nih.gov/BLAST
